# Parent and Health Care Provider Perceptions for Development of a Web-Based Weight Management Program for Survivors of Pediatric Acute Lymphoblastic Leukemia: A Mixed Methods Study

**DOI:** 10.2196/cancer.6680

**Published:** 2017-02-09

**Authors:** Sara Folta, Winnie Chang, Rachel Hill, Michael Kelly, Susan Meagher, W Paul Bowman, Fang Fang Zhang

**Affiliations:** ^1^ Friedman School of Nutrition Science and Policy Tufts University Boston, MA United States; ^2^ Smith College Northampton, MA United States; ^3^ Division of Pediatric Hematology and Oncology Cook Children's Medical Center Fort Worth, TX United States; ^4^ Department of Pediatrics School of Medicine Tufts University Boston, MA United States; ^5^ The Floating Hospital for Children Tufts Medical Center Boston, MA United States; ^6^ Department of Psychiatry School of Medicine Tufts University Boston, MA United States; ^7^ Department of Pediatrics University of North Texas Health Science Center Fort Worth, TX United States

**Keywords:** weight management, childhood cancer survivors, mixed methods

## Abstract

**Background:**

Survivors of pediatric acute lymphoblastic leukemia (ALL) may experience unhealthy weight gain during treatment, which has been associated with higher risk for chronic health issues.

**Objective:**

The purpose of this study was to obtain feedback on weight management in pediatric ALL survivors and on the content and implementation of a Web-based weight management program.

**Methods:**

Study participants included 54 parent survey respondents and 19 pediatric oncology professionals in 4 focus groups. Survey questions included report of child weight status and interest in participating in weight management programming at various time points. Pediatric oncology professionals were asked about the preferred topics and timing, as well as their role. Focus group data were analyzed by a multidisciplinary research team for common themes.

**Results:**

The mean age of survivors was 6.5 years. By parent report, 19% of children were overweight and 25% were obese. Preferred timing for weight management program participation was within 3 months of starting maintenance chemotherapy (23/53, 43%) or within 12 months after completion of all cancer treatments (18/53, 34%). Pediatric oncology professionals likewise considered the maintenance phase appropriate. They considered parenting to be an important topic to include and indicated that their most appropriate roles would be promotion and support.

**Conclusions:**

Parents and pediatric oncology professionals are interested in and supportive of early weight management in pediatric ALL survivors. Future research needs to identify strategies to integrate this into pediatric cancer care and to evaluate the feasibility and efficacy of these strategies.

## Introduction

As survival rates among children diagnosed with cancer are rising, there is increased attention toward longer term health outcomes [1]. Childhood cancer survivors are at higher risk of morbidity and mortality from a number of conditions, including cardiovascular dysfunction [2], for which obesity is an established risk factor. There is a high prevalence of obesity among children diagnosed with acute lymphoblastic leukemia (ALL) [3], the most common cancer diagnosed in children. Unhealthy weight gain tends to begin during early treatment [4] and may be associated with chemotherapeutic agents, which can impact energy intake directly through complex pathways and indirectly by affecting physical activity levels through muscle strength impairment [5,6]. However, the proportion of cases of obesity associated with cancer treatment exposure in adult survivors of childhood cancer is only 42% [1]. In addition to the effects of chemotherapy, cancer treatment may initiate a trajectory that results in survivors adopting behaviors that contribute to weight gain, including poor dietary habits and very low levels of physical activity [7,8]. This suggests that the time during which children are receiving cancer treatment and just beyond is a sensitive window for addressing unhealthy weight gain. There are very few studies of weight management programming specifically for pediatric ALL survivors [9,10] and none found in the literature that focus on introducing weight management earlier in treatment. Programs that target early onset of obesity are a priority for improving long-term health outcomes in this high-risk population.

For young children, behavior change may be appropriate through parent-centered approaches, whereby parents influence habits in the household environment [11]. In the general population, research has shown that parent feeding practices directly impact children’s lifelong dietary intake patterns and food relationships [12]. A previous qualitative study found that parents of pediatric cancer patients face unique stressors due to changes in appetite and weight during their child’s treatment, which may lead them to form permissive feeding habits (ie, allowing children a high degree of choice regarding amount and type of food consumed) despite knowledge of the unhealthy nature of the food they offer [13].

**Figure 1:**
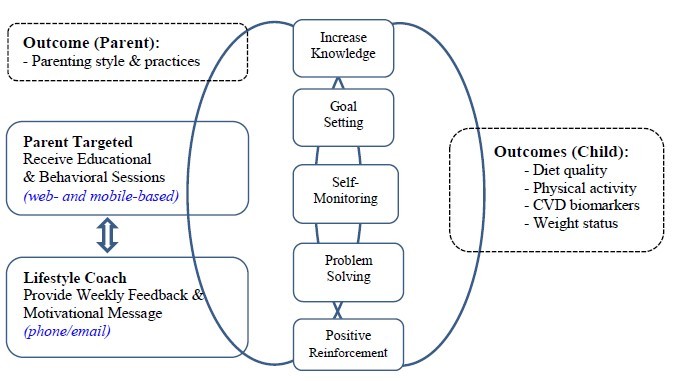
Healthy Eating and Active Living Program framework.

The Healthy Eating and Active Living (HEAL) program, currently in its pilot phase, was adapted from existing childhood obesity interventions studies [9,10,14-16]. The program was developed to provide resources to parents of ALL survivors and to assist them in setting actionable plans to transition their families into healthier eating and physical activity habits. In designing this program, it was important to address issues specific to pediatric cancer survivors’ experiences around unhealthy eating habits related to cancer treatment, such as food cravings [17] and changes in taste preference [18], as well as concerns such as the permissive feeding style that may develop. The program framework (Figure 1) and behavioral strategies are based on social cognitive theory [19]. A goal of this 12-week program was to provide the content missing from standard practice. It was designed to be Web-based and self-led and focused on the content areas of parenting, nutrition, and physical activity (see Textbox 1 for an outline of the curriculum).

**The 12-week curriculum of the Healthy Eating and Active Living program.**

Session 1: Get to Know the Program, Get to Know YOU

- The Healthy Eating and Active Living (HEAL) program
- Program focus areas: Parenting, nutrition, and physical activity
- Session evaluation: How on-target was this session?

Session 2: Effective Parenting

- Practice good communication skills
- Use appropriate parenting styles
- Set Specific, Measurable, Attainable, Realistic/Relevant, and Time-Bound (SMART) Plan: Develop action plans for effective parenting
- Session evaluation: How on-target was this session?

Session 3: Food and Nutrition Basics

- What are nutrition-rich foods? MyPlate Plan
- Choose healthy food portion size and read nutrition labels
- Fun activity with your child: Spotting the Block
- Set SMART Plan: Priority and confidence assessment on nutrition
- Session evaluation: How on-target was this session?

Session 4: Food and Nutrition Environment

- Food and nutrition environment assessment
- Eating together as a family and mindful eating
- Fun activity with your child: Grocery store scavenger hunt
- Set SMART Plan for my child and family: Nutrition
- Session evaluation: How on-target was this session?

HEAL Rewards: You Have Achieved BRONZE Status

Session 5: Physical Activity Basics

- The importance of physical activity
- Is physical activity safe for my child?
- Resistance training and bone health
- Set SMART Plan: Priority & confidence assessment on physical activity
- Session evaluation: How on-target was this session?

Session 6: Physical Activity Environment

- Physical activity environment assessment
- How to get the whole family to move
- Set SMART Plans for my child and family: Physical activity
- Session evaluation: How on-target was this session?

Session 7: Counting Energy In and Out

- Energy balance: Counting energy in and out
- Calorie intake and food portion size
- Energy expenditure and types of physical activity
- Fun activity with your child: Fun and fast circuit course activity
- Session evaluation: How on-target was this session?

Session 8: Barriers for Healthy Eating and Active Living

- Overcoming active living barriers
- Overcoming healthy eating barriers
- Identify my barriers
- Program goal: Revise my SMART plans
- Session evaluation: How on-target was this session?

*HEAL Rewards: You Have Achieved SILVER Status*

Session 9: Body Image

- Body image basics and beyond
- Body image environment assessment
- Minimize media impact activity
- Session evaluation: How on-target was this session?

Session 10: Emotional Eating and Stress Management

- Emotional eating
- Stress management
- Session evaluation: How on-target was this session?

Session 11: Beyond the Basics

- Satiety and energy density
- Strategies to eat less but healthy and eat less without being hungry
- Session evaluation: How on-target was this session?

Session 12: Move Forward with Healthy Habits

- Moving forward with healthy habits
- Keeping up with SMART plans
- Session evaluation: How on-target was this session?
- HEAL program final participant evaluation

HEAL Rewards: You Have Achieved GOLD Status

The aim of this study was use qualitative and quantitative methods to gain information to help iteratively refine the HEAL program while it was in the pilot phase. We therefore sought to understand perceptions on weight management generally in the pediatric ALL survivor population, to obtain feedback on the content of the HEAL program, and to gain insight on factors related to its implementation, such as the optimal timing and potential barriers.

## Methods

This study used a mixed methods approach, which involved both quantitative (survey) and qualitative (focus group) data collections methods. We describe the procedures for both methods below. A convergent mixed methods design [20] was used, whereby integration of findings occurred during the analysis phase.

### Survey With Parents for Weight Management in Pediatric ALL Survivors

Between September 1, 2014, and January 31, 2015, parents with children who attended the pediatric oncology clinics at 2 metropolitan medical centers in the United States, 1 in Texas and 1 in the Northeast region, were given a survey to complete anonymously. Parents were eligible if they had a child diagnosed with pediatric ALL who was between the ages of 3 to 11 years. To gain multiple perspectives on timing, the parent was eligible if the child was either receiving early treatment (chemotherapy prior to starting the maintenance phase), on maintenance therapy, or within 2 years of completion of all treatments. Surveys were distributed by the clinic receptionist or dietitian, who informed parents that participation was voluntary and would not affect clinical care. Participants placed the completed survey in an envelope and returned it to the receptionist, who then mailed them to the research team for data entry and analysis. The institutional review boards at both institutions approved this study.

The survey instrument included 13 items. Parents were asked about their child’s age, sex, current weight and height, type of cancer diagnosed, year at diagnosis, treatment status (on versus off treatment), and time of last treatment for those who had completed treatment.

To gain perceptions about timing, parents were also asked to indicate interest in participating in a 12-week online weight management program to help facilitate healthy eating and active living among their children at time points related to typical treatment milestones: (1) within 3 months after child starts maintenance therapy, (2) 3 to 6 months after child starts maintenance therapy, (3) 6 to 12 months after child starts maintenance therapy, (4) at least 12 months after child starts maintenance therapy, (5) within a year after child completes all treatments, (6) 1 to 2 years after child completes all treatments, (7) at least 2 years after child completes all cancer treatments, and (8) none of these points—not interested. Parents were asked to indicate obstacles that could prevent them from participating in an online weight management program for their child: lack of time, lack of Internet access, and/or lack of interest. They were also prompted to list any other obstacles not included in the survey.

Summary statistics were calculated using SAS version 9.3.1 (SAS Institute, Inc). Additionally, body mass index (BMI) was calculated using the standard approach (kg/m^2^). BMI *z*-score and BMI percentile were then calculated using the 2000 US Centers for Disease Control and Prevention (CDC) growth charts for children [21]. Weight categorizations were defined based on the current recommendations of the CDC [22].

### Focus Groups With Pediatric Oncology Professionals

A total of 4 focus groups with pediatric oncology professionals were conducted. One group was with a pediatric oncology team from the clinic at a northeastern medical center to gain perspectives from a full team involved with treatment and survivorship care. Email invitations were sent to all members of the team requesting their participation in the in-person focus group, held in May 2015. The remaining 3 groups were comprised of pediatric oncology dietitians who were members of the pediatric subunit of the Oncology Nutrition Dietetic Practice Group of the Academy of Nutrition and Dietetics, who were recruited between January 1, 2015, and May 30, 2015, via email. They represented 13 health care facilities in 9 states across the country. These groups were conducted via WebEx online conferencing.

All focus groups lasted approximately 1 hour and were moderated by the study principal investigator (FFZ), who was trained by another member (SCF) with expertise in qualitative methods. The focus group guide was developed by the principal investigator with input from the entire study team, including the qualitative methods expert. It was identical across all 4 groups, however the pediatric oncology dietitians received the HEAL curriculum for review prior to the groups while the pediatric oncology team was presented with the content during the focus group. Participants were asked about their experiences in working with pediatric ALL survivors around weight management, followed by questions on the content of the HEAL program and factors related to its implementation (format, delivery, timing, barriers, and their role). To help confirm that participant perceptions of key points were captured, just prior to concluding the moderator summarized main discussion points and asked for feedback on them. Participants received a $50 gift card for their participation.

Recordings of the focus group sessions were transcribed verbatim. Thematic analysis was undertaken by 5 members of the study team, who independently reviewed and themed the data by examining the transcripts for recurring patterns, implicit meaning, and negative evidence [23]. They then met and obtained consensus on themes; any areas of disagreement were resolved based on review of the transcripts. Data were then coded by theme by 1 research team member using Microsoft Excel (qualitative analysis software was not necessary based on the small number of groups). As a final step, the principal investigator and qualitative expert reviewed the codes and confirmed the themes. Supporting quotes for each theme were identified for each focus group.

## Results

### Characteristics

Characteristics of the pediatric ALL survivors, based on survey report by parents, are presented in Table 1. The average age of the children was 6.5 years, and 33 of the 54 children were male (61%). The mean reported age of diagnosis was 3.6 years, and mean time interval from diagnosis was 2.7 years. Children were in all 3 treatment stages. A slight majority of children were of normal weight, and the rest were overweight or obese per parent report of height and weight.

**Table 1:** Characteristics of pediatric acute lymphoblastic leukemia survivors in survey.

Characteristics		N=54^a^
Age, years, mean (SD)		6.5 (2.1)
**Gender, n (%)**		
	Male	33 (61)
	Female	21 (39)
Age at diagnosis, years, mean (SD)		3.6 (2.2)
Interval from diagnosis, years, mean (SD)		2.7 (1.3)
**Treatment status, n (%)^b^**		
	Currently receiving active treatment for cancer	16 (30)
	Completed active treatment and is now on maintenance chemotherapy	18 (34)
	Completed all cancer treatments	19 (36)
Body mass index percentile, mean (SD)		78.3 (21.5)
**Weight status, n (%)^c^**		
	Normal weight	27 (56)
	Overweight	9 (19)
	Obese	12 (25)
**Parents’ thoughts about child’s weight status, n (%)**		
	I would like to help my child gain weight	1 (2)
	I would like to help my child continue to maintain a healthy weight for his/her age	42 (78)
	I would like to help my child lose weight	8 (15)
	I don’t think about my child’s weight	3 (6)
**Parents’ interest in participating in weight management program at various time points during and after treatment, n (%)**		
	Within 3 months after child starts maintenance therapy	23 (43)
	At 3 to 6 months after child starts maintenance therapy	11 (21)
	At 6 to 12 months after child starts maintenance therapy	15 (28)
	At least 12 months after child starts maintenance therapy	15 (28)
	Within a year after child completes all treatments	18 (34)
	At 1 to 2 years after child completes all treatments	10 (19)
	At least 2 years after child completes all treatments	8 (15)
	None of these points—not interested	5 (9)

^a^Percentages were calculated based on the number of respondents who provided answers to specific questions.

^b^Active treatment refers to the period from the start of treatment until the start of maintenance chemotherapy.

^c^Weight status was defined based on BMI *z*-score or percentile using the 2000 Centers for Disease Control and Prevention growth charts for children. Normal weight was defined as BMI *z*-score = −1.645 to 1.035 (5th-84.9th percentile), overweight as BMI *z*-score = 1.036-1.644 (85th-94.9th percentile), and obese as BMI *z*-score ≥ 1.645 (≥ 95th percentile).

The focus group with the pediatric oncology team was comprised of 3 pediatric oncologists, 3 nurses, 1 social worker, and 1 child life specialist. Participants in the 3 remaining groups were 13 oncology dietitians and 1 social worker, and group size ranged from 3 to 6 participants.

### Importance of Weight Programming

Both the quantitative (survey) and qualitative (focus group) results support a need for weight management programming for pediatric ALL survivors. On the surveys, the majority of parents (43/54, 78%) indicated that they would like to help their child continue to maintain a healthy weight for his/her age; 8/54 (15%) indicated that they would like to help their child lose weight (Table 1). In the focus groups, dietitians and pediatric oncology team members reported observing significant weight gain in pediatric ALL patients. They described weight gain as tending to start during treatment, with behaviors that contributed to weight gain becoming habitual into survivorship (Table 2).

**Table 2:** Topic areas, major themes, and representative quotes from focus groups with pediatric oncology professionals.

Topic	Themes	Representative quotes
Program content	There is a need for weight management programming overall. The parenting component of the program is critical. The nutrition information could be improved to be simpler, shorter, and more practical.	“. . . a lot of questions that I get even in the hospital really have to do a lot with the parenting and normal feeding struggles that families have. . . I really like that you start out with that because I think it helps parents gain some skills.” [Pediatric oncology dietitian] “I think it’s just, the parents always go back to ‘Well my kid has cancer so they’re going to do whatever they want.’ It’s just the permissive parenting that we see all the time. . .” [Pediatric oncology nurse] “A lot of my patients, even if they may read things online and have a lot of access to nutrition-related information, their understanding of nutrition is very basic and this is high-level nutrition information that’s being presented.” [Pediatric oncology dietitian]
Program implementation: timing	Starting programming during treatment would be ideal but is not feasible. The start of maintenance treatment is the most appropriate time to begin programming. Anticipatory guidance could be given at earlier stages of treatment.	“When you’re looking at a leukemia [patient] maybe maintenance therapy is a good time to intervene because they’re getting treated more like a healthy kid.” [Pediatric oncology dietitian] “I always try at least even in the initial stages of treatment to give a little anticipatory guidance. . . some information upfront so that they have it, but not really getting into any serious counseling until maintenance.” [Pediatric oncology dietitian]
Program implementation: format	The Web-based, self-directed format was considered useful and appropriate. More audio and/or video would break up the content and could improve accessibility. The program should not extend beyond 12 weeks.	“I personally really like the computer or Web-based delivery, because I think you have an opportunity to reach more people that have a different schedule or different families that are split apart. . . And also just the fact that they can do it at any time of day is going to make it able to be available to some people that wouldn’t, based on work schedule or travel.” [Pediatric oncology dietitian]
Program implementation: barriers	Time and Internet and computer accessibility could pose challenges for families.	“Just because as a parent, whether or not they even have a job, just having 20 minutes just to themselves, might be hard to find that.” [Pediatric oncology dietitian]
Role of pediatric oncology professionals	The most appropriate role for pediatric dietitians would be to provide an introduction and support. Information about program-related nutrition and physical activity goals could be incorporated into patients’ charts and records.	“. . . with maintenance, we generally only see our patients once a month. . . it’s outpatient per request . . . So I wouldn’t even be the person to see them and remind them.” [Pediatric oncology dietitian] “I could see it as just telling them to reach out to me when they have questions.” [Pediatric oncology dietitian] “And if [physicians are] really supporting the program, then we’ll probably find some way to incorporate it into maybe [electronic medical record system].” [Pediatric oncology dietitian]

### Program Content

Findings from the focus groups included several themes regarding the content of the HEAL program. A major theme expressed in all groups was that the content on effective parenting is critical for successful weight management, especially since permissive feeding practices were a likely contributor to weight gain in the children. Another theme was that addressing parents’ fears about physical activity safety would be especially helpful for this population. Several themes emerged related to aspects of the program content that could be improved. Across the groups with dietitians, the nutrition information was perceived as too advanced for parents (eg, content on weight status and energy density). The clinical oncology team discussed the likely benefit of adding very practical nutrition advice, such as tips for food shopping or kid-friendly recipes. There was also concern that too much information was presented overall, and it was suggested that the content could be condensed to make it more reader-friendly and easier to comprehend.

### Program Implementation

#### Timing

Data on optimal timing of the program were obtained from both the surveys and the focus groups. The points parents selected on the survey to be most of interest for weight management programming were within 3 months of starting maintenance therapy (23/54, 43%) and within a year of completing all treatments (18/54, 34%). Parents’ selection of preferred timing did not differ by current treatment status of their child (data not shown). In the focus groups with pediatric dietitians, a theme was that information about healthy eating and physical activity would ideally be delivered during treatment, before negative habits could form. However, this was unlikely to be feasible since, in their perception, parents would be less receptive to information not directly related to treatment, and unhealthy eating might be tolerated in an effort to maintain the child’s weight. There was therefore consensus across the groups that the most appropriate time to start the actual weight management programming would be around the start of maintenance, as the children are transitioning back into their regular lifestyles. However, in both types of focus groups, it was mentioned that providing anticipatory guidance around nutrition and physical activity education at earlier treatment stages may be helpful for parents.

#### Format

In terms of format, pediatric oncology dietitians felt that the Web-based, self-directed delivery of the HEAL program would be useful for parents since it allows for flexibility with time and pace of completion. They indicated that they also preferred this format to an in-person or telephone-based counseling method of delivery since as dietitians they are limited in their ability to deliver information, due to infrequent contact with patients and general time constraints. In each of the groups, there was at least 1 suggestion to convert the program from its text-heavy format to include more audio and/or video presentations, for example, as a narrated slideshow. In 1 of the groups, it was suggested that audio features may improve the accessibility of the content for families in which there may be low literacy. As for the length of the program, there was agreement among the dietitians that the program should not extend longer than 12 weeks and that sessions should be kept brief (around 20 minutes), otherwise parents would likely lose interest.

#### Barriers

Both quantitative and qualitative methods provided information on barriers to participation in the program. Among the 41 participants who answered the question about barriers on the survey, 29 (71%) of parents selected lack of time and 6 (15%) of parents selected lack of Internet access as obstacles that could prevent them from participating. In the focus groups, in response to an open-ended question about barriers, pediatric oncology dietitians also mentioned both time and Internet and computer accessibility as potential challenges for families. However, in 2 groups it was suggested that accessibility on portable devices (such as smartphones and tablets) would mitigate this since most parents have access to these devices. Some pediatric oncology dietitians also mentioned that eventual translation of the program into Spanish would be important to widening the reach of the program.

### Role of Pediatric Oncology Professionals

In the focus groups, several themes emerged related to the role of the pediatric oncology professionals in weight management programming. Across groups, all were supportive of the HEAL program. The dietitians indicated that appropriate roles would be to introduce it to patients and to support it by scheduling an in-person or phone meeting with parents when they had specific questions. Pediatric oncology professionals suggested the possibility of incorporating information about nutrition and physical activity goals and behaviors that parents inputted as part of the program into patients’ charts and records, to be reviewed during hospital visits. In 1 focus group, the dietitians suggested that this type of integration may make parents feel more accountable for their progress in the program and boost adherence.

## Discussion

### Principal Findings

In this mixed methods study, data were obtained from both parents and health care providers who work closely with pediatric ALL patients in addressing behaviors and issues related to weight management. Taken together, the data from these sources confirm a need and desire for weight management programming for pediatric ALL survivors. Health care professionals indicated the importance of the content on effective parenting for successful weight management. They suggested that the nutrition content should be simple and include practical tips. Results indicate that the most appropriate and feasible time to start the weight management programming is around the start of maintenance therapy, as the children are transitioning back into their regular lifestyles. The Web-based, self-directed delivery of the program was viewed favorably since it allows for flexibility with time and pace of completion, but health professionals suggested that there may be barriers related to health literacy and access to technology. They indicated that their appropriate role would be to introduce the program and to support its use.

Most parents indicated that that they would like to help their children maintain a healthy weight or lose weight. Consistent with the literature [4], pediatric oncology professionals, both the team and the dietitians, have observed weight gain in this population, which they perceived as related to behaviors within families that become habitual during treatment and therefore continue into survivorship. Permissive parenting related to food that develops during treatment was raised as a major contributing factor and suggested a need for guidance on reexamining parenting practices as children transition into survivorship. The HEAL program content on effective parenting was therefore viewed as essential.

Several of these findings are similar to 1 other mixed methods study designed to inform an obesity intervention for pediatric cancer survivors [10]. In that study, pediatric oncology professionals likewise noted behaviors that develop during treatment that then become habitual and difficult to reverse during the transition from treatment. Parents noted the changes in habits that occurred during treatment and, as in this study, expressed interest in weight management programming.

Despite this interest, results from the parent surveys suggest a disconnect between perceived and actual weight status of the children, with a significant number indicating that their children are at a healthy weight for age despite BMI *z*-scores that place children in overweight and obese categories. This mirrors inaccurate perceptions that exist in the general population [24] and suggests that clinicians may have an important role to play in reviewing growth charts and discussing weight.

In focus groups, the Web-based format was perceived favorably since it would be convenient for parents and have a wide reach. It may also be more feasible than in-person counseling due to time constraints among parents, who indicated that time would be a major barrier to participation. Pediatric oncology professionals suggested prioritizing succinctness over comprehensiveness to improve adherence to the program. The program content has since been shortened based on this feedback. Pediatric oncology professionals indicated that their role would entail aiding in the promotion and introduction of the HEAL program and possibly also discussing aspects of it, such as behavioral goals, during clinical visits.

Oncology professionals suggested that a Web-based program should use interactive features and multimedia to engage readers. They also suggested presenting information in smaller sections. Based on this feedback, a narrated slideshow was added to each session of the curriculum for the pilot program as an alternative way to access content. It was further suggested that audio may be beneficial for reaching low-literacy audiences. However, while providing audio may help in making the content more accessible to parents with limited reading ability, inherent complexity of the underlying concepts could still serve as a major barrier to parents’ ability to comprehend, evaluate, and use the information [25]. This issue merits additional attention as the program continues to be developed.

### Strengths and Limitations

This study had several strengths and limitations. Parents whose children are in treatment for cancer are challenging to reach since families are essentially in crisis. By using mixed methods, salient data were feasibly obtained quantitatively from parents and validated and extended with qualitative data from professionals who interact most with parents on these issues. Surveys were conducted with a relatively small number of parents from 2 pediatric cancer clinics and may not generalize. Focus groups were likewise conducted with convenience samples: the pediatric oncology team was geographically located at the researchers’ home institution, and the dietitians were accepted as they responded to recruitment. However, the dietitians were part of a professional practice group and represent pediatric oncology clinics across the United States. The survey was conducted anonymously in the clinics, and the response rate is unknown. An additional limitation of the anonymous survey is that parent reports of children’s heights and weights were not corroborated by clinical data. However, the BMI distribution observed in this sample is consistent with measured values from a previous study conducted by the authors with a similar cohort [26]. To keep the survey to 2 pages, parents were not asked about ethnicity or socioeconomic status. However, the clinics where the surveys occurred treat a diverse range of families. Finally, parents were not asked about the delivery format or content of the program in this formative stage. Instead, detailed feedback is being obtained as parents complete a pilot version of the program.

### Conclusion

This study found that parents and pediatric oncology professionals were interested in and supportive of weight management programming for pediatric ALL survivors. They provided valuable input on the content and implementation of this type of program. Future studies will involve testing the HEAL program for feasibility and effectiveness. Clinicians are likely to play an important role by offering anticipatory guidance and promoting and supporting such programming.

## Abbreviations

ALL: acute lymphoblastic leukemia
BMI: body mass index
CDC: Centers for Disease Control and Prevention
SMART: Specific, Measurable, Attainable, Realistic/Relevant, and Time-Bound
